# Supplementary material for: Suppression of hesA mutation on nitrogenase activity in Paenibacillus polymyxa WLY78 with the addition of high levels of molybdate or cystine
Source: PeerJ. 2019 Feb 1;7:e6294. doi: 10.7717/peerj.6294 (PMC6361004; doi:10.7717/peerj.6294)
Supplement: Supplemental Information 2 [file peerj-07-6294-s002.docx]

**Table S1.** Strains used in this study.

| **Strains** | **Genotype or phenotype** | **Source** |
| --- | --- | --- |
| *P. polymyxa* WLY78 | N_2_-fixing bacterium | Lab stock |
| *E.coli* JM109 | *recA1, endA1, gyrA96，thi-1，hsdR17, supE44, relA1,* Δ（*lac-proAB*）*/*F’[*traD36, proAB+, lacIq, lacZ*Δ*M15*] | Takara Bio, cat. no. 9022 |
| *E. coli* 78-7 | A [derivative](http://www.baidu.com/link?url=u_6LWc7ccWZb9DlDQutSdNvK3sR2lg7gWg6FQc3NHME3MvckpN6MiGHSKfN9jBhu1bJbbmlujtRZn2P9Cl_dvxdpQneWb7u4HArRRw585Ua) of *E. coli* JM109 carrying a *nif* gene cluster (*nifBHDKENXhesAnifV*) from *P. polymyxa* WLY78 | Lab stock |
| Δ*hesA* | A *hesA* delection mutant strain of *P. polymyxa* WLY78; Chl^r^ | This study |
| Δ*hesA* (pHY300PLK-*hesA*) | A complementation strain which is a *ΔhesA* mutant carrying *P. polymyxa hesA* in plasmid pHY300PLK-*hesA* (P*nif*+*hesA* gene); Tet^r^ | This study |
| Δ*hesA* (pHY300PLK-*nifQ*) | A complementation strain which is a *ΔhesA* mutant carrying *K. oxytoca* *nifQ* in plasmid pHY300PLK-*nifQ* (P*nif*+*nifQ* gene); Tet^r^ | This study |
| Δ*hesA* (pHY300PLK-*moeB*) | A complementation strain which is a *ΔhesA* mutant carrying *E. coli moeB* in plasmid pHY300PLK-*moeB* (P*nif*+ *moeB* gene); Tet^r^ | This study |
| D-O (Δ*hesA*) | A derivative of *E. coli* JM109 carrying a *Paenibacillus* *nif* gene cluster (*nifBHDKENXhesAnifV*) with *hesA* being deleted in plasmid pHY300PLK; Amp^r^, Tet^r^ | Lab stock |
| D-O (pBluescript II SK (+)-*hesA*) | A complementation strain which is a D-O (Δ*hesA*) strain carrying *P. polymyxa hesA* in plasmid pBluescript II SK (+)-*hesA*; Amp^r^, Tet^r^, Chl^r^ | This study |
