# Supplementary material for: Suppression of hesA mutation on nitrogenase activity in Paenibacillus polymyxa WLY78 with the addition of high levels of molybdate or cystine
Source: PeerJ. 2019 Feb 1;7:e6294. doi: 10.7717/peerj.6294 (PMC6361004; doi:10.7717/peerj.6294)
Supplement: Supplemental Information 3 [file peerj-07-6294-s003.docx]

**Table S2.** Sequences of primers used in this study.

| **Gene name** | **Forward primer (5' - 3')** | **Reverse primer (5' - 3')** | **Target** |
| --- | --- | --- | --- |
| Left arm | Up-F (acgatgcgtccggcgtagaggatccGATTCAGCCTGCCAGATG) | Up-R (taatttttttaaggcaCAGTTGTACCATTCGTCCAC) | For cloning the plasmid  pRN5101-Cm of the *hesA* delection mutant strain |
| Chl | Chl-F (ggtacaactgTGCCTTAAAAAAATTACGC) | Chl-R (ccgactcatcAAGAATTAATTCACTGGCC) | For cloning the plasmid  pRN5101-Cm of the *hesA* delection mutant strain |
| Right arm | Down-F (aattaattcttGGAACGGGAGGGGATACA) | Down-R (cgcaaaagacataatcgataagctTAATACTGCAGCCAGCGTC) | For cloning the plasmid  pRN5101-Cm of the *hesA* delection mutant strain |
| *nif* promoter | Pnif-F (CCGGAATTCGCGGAGACTATTTCCCAAA) | Pnif-R (CACGGATCCCTCCTCTCTACGTTATATTC) | For cloning the *nif* cluster promoter from *P. polymyxa* WLY78 to pHY300PLK |
| *hesA* | hesA-F (CACGGATCCAAAATCCTGCATGCTGAGGA) | hesA-R (CGCAAGCTTTCACAAATATGCAACCGAC) | For cloning the *P. polymyxa hesA* gene to pHY300PLK |
| *nifQ* | nifQ-F (AAAGGATCCAAGGGGGTCGCCTGATGCC) | nifQ-R (CGCAAGCTTTTGTTTGCAGCGCCCCCTTC) | For cloning the *K.oxytoca nifQ* gene to pHY300PLK |
| *moeB* | moeB-F  (CAAGGATCCGGAGGCCTGTAATGGCGGA) | moeB-R  (CGCAAGCTTCCGGACGTTCTGCATCCTCA) | For cloning the *E. coli moeB* gene to pHY300PLK |
| P*nif-hesA* | Pnif-F (CCGGAATTCGCGGAGACTATTTCCCAAA) | hesA-R (CGCAAGCTTTCACAAATATGCAACCGAC) | For cloning the *P. polymyxa hesA* gene to pBluescript II SK (+) |
| Chl | Cm (pPR9TT)-F(KpnI)  (ctcggtaccATTAGCAGAGCGAGGTATG) | Cm (pPR9TT)-R (KpnI) (ctcggtaccGCGGAACCCCTATTTGTTT) | For cloning the chloramphenicol resistance gene from plasmid pPR9TT  to pBluescript II SK (+) |
| 16S rDNA | 16S-F (TTTGTCGTCAGCTCGTGTTCGTG) | 16S-R  (ATCCCCACCTTCCTCCGGTTTG) | qRT-PCR for a control |
| *nifV* | nifV-F  (GCCATAGCTGCCCGTATAGA) | nifV-R  (CTCCAGCGCGGTATTACCTG) | qRT-PCR for *nifV* |
