# Supplementary material for: Suppression of hesA mutation on nitrogenase activity in Paenibacillus polymyxa WLY78 with the addition of high levels of molybdate or cystine
Source: PeerJ. 2019 Feb 1;7:e6294. doi: 10.7717/peerj.6294 (PMC6361004; doi:10.7717/peerj.6294)
Supplement: Supplemental Information 4 [file peerj-07-6294-s004.docx]

**Table S3.** Plasmids used in this study.

| **Strain/Plasmids** | **Relevant characteristic(s)** | **Reference or source** |
| --- | --- | --- |
| pRN5101 | A 8.09 kb shuttle vector between *B. subtilis* and *E. coli* with a temperature-sensitive region; Amp^r^, Em^r^ | Gifted by associate professor Yanqin Ding from Shandong Agricultural University |
| pPR9TT | A broad-host range *lacZ* promoter probe vector, RK2 replicon; Amp^r^, Chl^r^ | Lab stock |
| pRN5101-Cm | A pRN5101 derivative carrying homologous arms of *hesA* gene and chloramphenicol resistance cassette; Amp^r^, Em^r^, Chl^r^ | This study |
| pHY300PLK | A 4.87 kb shuttle vector between *B. subtilis* and *E. coli*; Amp^r^, Tet^r^ | Sangon Biotech Co., cat. no. 3060 |
| pHY300PLK-78 | A pHY300PLK derivative, carrying *P*. *polymyxa* WLY78 *nif* cluster under the control of its own *nif* promoter; Amp^r^, Tet^r^ | Lab stock |
| pHY300PLK-*hesA* | A pHY300PLK derivative，carrying *P. polymyxa hesA* gene driven by *P. polymyxa* WLY78 *nif* promoter; Amp^r^, Tet^r^ | This study |
| pHY300PLK-*nifQ* | A pHY300PLK derivative，carrying *K. oxytoca* *nifQ* gene driven by *P*. *polymyxa* WLY78 *nif* promoter; Amp^r^, Tet^r^ | This study |
| pHY300PLK-*moeB* | A pHY300PLK derivative，carrying *E. coli* *moeB* gene driven by *P*. *polymyxa* WLY78 *nif* promoter; Amp^r^, Tet^r^ | This study |
| pBluescript II SK (+) | ColE1; Amp ^r^ | Lab stock |
| pBluescript II SK (+)-Cm | A pBluescript II SK (+) derivative, carrying chloramphenicol resistance cassette; Amp^r^, Chl^r^ | This Study |
| pBluescript II SK (+)-*hesA* | ＡpBluescript II SK (+) derivative, carrying chloramphenicol resistance cassette and *P. polymyxa hesA* gene driven by *P. polymyxa* WLY78 *nif* promoter; Amp^r^, Chl^r^ | This Study |
